# Supplementary material for: ProteinShader: illustrative rendering of macromolecules
Source: BMC Struct Biol. 2009 Mar 30;9:19. doi: 10.1186/1472-6807-9-19 (PMC2672931; doi:10.1186/1472-6807-9-19)
Supplement: Additional file 1 — ProteinShader program without source code. This compressed file contains the complete ProteinShader program including associated libraries, but no source code. A README.txt file gives an overview of the ProteinShader distribution, and the index.html file in the help subdirectory has directions on getting started with the program as well as a set of tutorials. [file 1472-6807-9-19-S1.zip › ProteinShader-beta-0_9_4-binary/help/api/org/proteinshader/gui/package-summary.html]

org.proteinshader.gui (ProteinShader API)


|  |  |  |
| --- | --- | --- |
| **Overview** \| **Package** \| Class \| **Use** \| **Tree** \| **Deprecated** \| **Index** \| **Help** | |  |
| **PREV PACKAGE**   **NEXT PACKAGE** | **FRAMES**    **NO FRAMES**     **All Classes** |  |


---

## Package org.proteinshader.gui

Holds all of the Swing GUI components and their associated listeners,
including class Renderer, which is registered as a listener for the GLCanvas
object that is used a drawing surface.

**See:**
  
          **Description**

| **Interface Summary** | |
| --- | --- |
| **Mediator** | This interface specifies the methods for the single central mediator object that allows communication between objects of the gui, the data structure, and the renderer. |

| **Class Summary** | |
| --- | --- |
| **MediatorImpl** | Serves as the central mediator central object that allows communication between objects of the gui, the data structure, and the renderer. |
| **ProteinShader** | Opens the main GUI window for the ProteinShader program. |
| **ProteinShaderGUI** | Provides the main GUI window for the ProteinShader program. |
| **ProteinShaderSplasher** | Opens a splash screen, ProteinShaderLogo.png, while the ProteinShaderGUI is in the process of starting up. |
| **Renderer** | Knows how to render a protein image onto a GLCanvas. |

## Package org.proteinshader.gui Description

Holds all of the Swing GUI components and their associated listeners,
including class Renderer, which is registered as a listener for the GLCanvas
object that is used a drawing surface.
